# The Ethanolic Extract of *Taiwanofungus camphoratus* (*Antrodia camphorata*) Induces Cell Cycle Arrest and Enhances Cytotoxicity of Cisplatin and Doxorubicin on Human Hepatocellular Carcinoma Cells

**DOI:** 10.1155/2015/415269

**Published:** 2015-10-18

**Authors:** Liang-Tzung Lin, Chen-Jei Tai, Ching-Hua Su, Fang-Mo Chang, Chen-Yen Choong, Chien-Kai Wang, Cheng-Jeng Tai

**Affiliations:** ^1^Department of Microbiology and Immunology, School of Medicine, College of Medicine, Taipei Medical University, Taipei 11031, Taiwan; ^2^Department of Obstetrics and Gynecology, School of Medicine, College of Medicine, Taipei Medical University, Taipei 11031, Taiwan; ^3^Department of Chinese Medicine, Taipei Medical University Hospital, Taipei 11031, Taiwan; ^4^Traditional Herbal Medicine Research Center, Taipei Medical University Hospital, Taipei 11031, Taiwan; ^5^Taipei Medical University, Taipei 11031, Taiwan; ^6^School of Dentistry, College of Oral Medicine, Taipei Medical University, Taipei 11031, Taiwan; ^7^Division of Hematology and Oncology, Department of Internal Medicine, Taipei Medicine University Hospital, Taipei 11031, Taiwan; ^8^Department of Internal Medicine, School of Medicine, College of Medicine, Taipei Medical University, Taipei 11031, Taiwan

## Abstract

*Taiwanofungus camphoratus* (synonym *Antrodia camphorata*) is a widely used medicinal fungus in the folk medicine of Taiwan with several pharmacological features such as anti-inflammatory, liver protection, antihypertensive, and antioxidative activities. The ethanolic extract of *T. camphoratus* (TCEE) which contains abundant bioactive compounds including triterpenoids and polysaccharides also has antitumor effects in various human cancer cell lines. The aims of this study are to clarify the antitumor effects of TCEE on human hepatocellular carcinoma cells and also evaluate the combination drug effects with conventional chemotherapy agents, cisplatin and doxorubicin. In the present study, the TCEE treatment induced cell cycle arrest and suppressed cell growth on both Hep3B and HepJ5 cells. Expression of cell cycle inhibitors, P21 and P27, and activation of apoptosis executer enzyme, caspase-3, were also induced by TCEE. In combination with the chemotherapy agents, TCEE treatment further enhanced the tumor suppression efficiency of cisplatin and doxorubicin. These results together suggested that TCEE is a potential ingredient for developing an integrated chemotherapy for human liver cancer.

## 1. Introduction

Liver cancer is one of the most common cancer types worldwide and has a particularly high incidence in sub-Saharan Africa and Eastern Asia regions [1]. Mortality of liver cancer remains high because of the difficulty of early diagnosis, high recurrence, and unavailability of potentially curative therapies such as surgical resection and liver transplantation [2]. Most advanced and recurrent cases therefore will receive systemic chemotherapies as the alternative approach. Chemotherapy agents such as doxorubicin, cisplatin, and 5-fluorouracil are the primary choices for treating liver cancer cases but the response rate and overall survival remained poor [3, 4]. Although recent targeted cancer therapy agents such as sorafenib demonstrate an improved clinical outcome in advanced liver cancer cases [5], the overall mortality rate of liver cancer still exceeds 90% worldwide [1]. The development of alternative or adjuvant treatments to improve the clinical outcome of the conventional therapy for liver cancer is therefore in urgent need.

The use of complementary and alternative medicine has become a very popular option to support the conventional therapy in many countries [6–8]. For example, many herbal formulas and remedies based on the traditional Chinese medicine are well accepted among cancer patients with Chinese background [9–11]. In Taiwan, a rare* Ganoderma*-like fungus,* T. camphoratus* (synonym* Antrodia camphorata*), is used as a medial component for health purpose by the aboriginal Taiwanese to modulate the immune system [12, 13] or improve liver function [14, 15]. In recent years, the ethanolic extract from fruity body of* T. camphoratus* (TCEE) which contains abundant triterpenoids and polysaccharide is widely used as a nutrient supplement in Taiwan. This TCEE also demonstrates antitumor properties such as the induction of cell cycle arrest and activation of apoptosis on human colon, lung, melanoma, osteosarcoma, and pancreatic cancer cells [16–19]. Moreover, treatment with TCEE is found to enhance the cytotoxic effects of amphotericin B in human colon cancer cell both in vitro and in vivo [17]. In contrast, the antitumor effects and related biological mechanism of TCEE as well as the combination drug effects with conventional chemotherapy agents remain unclear particularly in human hepatocellular carcinoma cells.

The aims of this preclinical study are to evaluate the capability of TCEE to suppress human hepatocellular carcinoma cells and clarify the related antitumor effects. Furthermore, the combined drug effects of TCEE with conventional chemotherapy agents, cisplatin and doxorubicin, were also analyzed to clarify whether TCEE enhances or antagonizes the cytotoxicity of the selected chemotherapy agents in hepatocellular carcinoma cells. This study may provide meaningful information to understand if TCEE is a potentially beneficial ingredient to integrate with cisplatin and doxorubicin for treating liver cancer.

## 2. Materials and Methods

### 2.1. Preparation of TCEE

The solid-state cultivated fruit body of* T. camphoratus*, provided by TWHerb Biomedical Co., Ltd. (Hsinchu, Taiwan), which contains 15 to 20% triterpenoids and 1 to 2% polysaccharides, was pulverized for further ethanolic extraction. Briefly, the 20 g pulverized raw materials were suspended into 95% ethanol (1 : 40 w/v) for an overnight incubation in room temperature. This ethanol extract was then filtered by 0.2 *μ*m Minisart filter (Sartorius, Gottingen, Germany) to remove insoluble debris and lyophilized. The recovery rate of lyophilized ethanol extract of* T. camphoratus* was 16.8%. The final concentration of ethanolic extract of* T. camphoratus* (TCEE) was adjusted to 1 g pulverized fruit body of* T. camphoratus* (168 mg lyophilized ethanol extract powder) per mL ethanol and stored at −20°C before experiment.

### 2.2. Cell Culture and Treatments

Human hepatocellular carcinoma cell lines Hep3B and HepJ5 were used for examining the antitumor effects of TCEE. Hep3B is a hepatocellular carcinoma cell with P53 deficiency [20], whereas HepJ5 cells are more malignant and drug resistant with the overexpression of survivin and glucose regulated protein-78 (GRP-78) [21, 22]. Both of them were purchased from the Bioresource Collection and Research Center (Hsinchu, Taiwan). Hep3B and HepJ5 cells were cultured in Dulbecco's modified Eagle's medium (Gibco, Grand Island, NY, USA) and fetal bovine serum (Gibco, Grand Island, NY, USA) with the mixture of 100 U/mL of penicillin and 100 *μ*g/mL of streptomycin (Invitrogen Life Technologies, Carlsbad, CA, USA) at 37°C in a 5% CO_2_ humidified incubator.

To evaluate the antiproliferation effect of TCEE on Hep3B and HepJ5 cells, cells were seeded into a 96-well plate at the concentration of 5 × 10^3^ overnight and then treated with 0 to 10 mg/mL TCEE for 48 hr. To evaluate the combination drug effects of TCEE with cisplatin and doxorubicin, Hep3B and HepJ5 cells were seeded in the same cell density, incubated overnight, and treated with 0 to 20 *μ*M cisplatin or 0 to 5 *μ*M doxorubicin in combination with 0.2 or 0.5 mg/mL TCEE for 48 hr. Both cisplatin and doxorubicin were purchased from Sigma-Aldrich (St. Louis, MO, USA). Microscope observation was performed by Nikon Eclipse TS100 optical microscope (Nikon Instruments, Melville, NY, USA) and photographed at 100x magnification before cell viability assay. Cell viability was then determined by using the 3-(4,5-dimethylthiazol-2-yl)-2,5-diphenyltetrazolium bromide (MTT, Bio Basic Canada Inc., Ontario, Canada) assay.

### 2.3. Cell Cycle Analysis

Hep3B and HepJ5 cells were seeded as 5 × 10^5^ cells per 6 cm dish, incubated overnight, and then treated with 1 or 2 mg/mL TCEE, respectively, for 24 hr. After treatment, cells were washed by phosphate buffered saline (PBS) and harvested by 0.05% trypsin-ethylenediaminetetraacetic acid (Life Technologies, Carlsbad, CA, USA). Cell pellets were collected by centrifuge and resuspended in 1 mL PBS with 4 mL 75% ethanol in −20°C overnight for cell fixation. Fixed cells were centrifuged and washed by 5 mL PBS in room temperature. Before analysis, cell suspensions were mixed with 1 mL propidium iodide buffer alone (RNase A 0.2 mg/mL, triton X-100 0.1%, and 20 *μ*g/mL propidium iodide, PI) or in combination with the FITC-labelled annexin V binding buffer (Invitrogen, Life Technologies, Carlsbad, CA, USA) in room temperature for 15 min staining. Stained cells were finally measured by the FACScan flow cytometer (BD Biosciences, CA, USA) and analyzed by the CellQuest software (BD Biosciences, CA, USA).

### 2.4. Western Blotting Analysis

Hep3B and HepJ5 cells were seeded as 5 × 10^5^ cells per 6 cm dish for an overnight incubation and treated with 0.5 or 1.0 TCEE for 48 hr. Cells were then washed by ice-cold PBS and harvested by the cell lysis buffer containing 150 mM NaCL, 50 mM Tris-HCL (pH 7.5), 1% NP-40, 0.5% deoxycholate, 0.1% SDS, 1 mM PMSF, 10 *μ*g/mL of leupeptin, and 100 *μ*g/mL of aprotinin to obtain the cell lysates. The total protein concentrations of the cell lysates were finally determined by the Bio-Rad protein assay kit (Bio-Rad Laboratories, Hercules, CA, USA) and equalized for the sodium dodecyl sulfate polyacrylamide gel electrophoresis. Separated protein was then transferred to a polyvinylidene fluoride membrane (Pall Corp., Port Washington, NY, USA) and detected by selected primary antibodies. Anti-survivin (Abcam, Cambridge, MA, USA) and anti-GRP-78 (Santa Cruz Biotechnology, Dallas, TX, USA) were used for identifying overexpression of survivin and GRP-78 on HepJ5 cells, whereas anti-P21 and anti-P27 (Millipore, Billerica, MA, USA) and anti-caspase-3 (Cell Signaling Technology, Danvers, MA, USA) were used for evaluating induction of cell cycle inhibitors and activation of apoptotic protein, caspase-3. Anti-glyceraldehyde 3-phosphate dehydrogenase (GAPDH, Abfrontier, Seoul, South Korea) served as control protein. The immunoreactivity was further analyzed by the WesternBright western blotting detection kit (CA, USA), and the intensity of immunoreactive bands was quantified by the ImageJ software (National Institutes of Health, Bethesda, MD, USA).

### 2.5. Statistical Analysis

In the presented study, one-way ANOVA was used for analyzing the dose-dependent effect of TCEE on Hep3B and HepJ5 cells, and Student's *t*-test was used for analyzing difference between groups. Both one-way ANOVA and Student's *t*-test were performed by using the SPSS software (SPSS Inc., Chicago, IL, USA). The half-maximal inhibitory concentration (IC_50_) and the combined drug index (CDI) of TCEE with conventional agents, cisplatin and doxorubicin, were analyzed by using the CalcuSyn software (Biosoft, Cambridge, UK), which is based on Chou-Talalay median effect method [23]. The obtained CDI value indicates additive (=1), antagonist (>1), or synergistic (<1) effects [24–26].

## 3. Results

### 3.1. Cell Growth Inhibition by TCEE Treatment on Human Hepatocellular Carcinoma Cells

**Figure 1:**
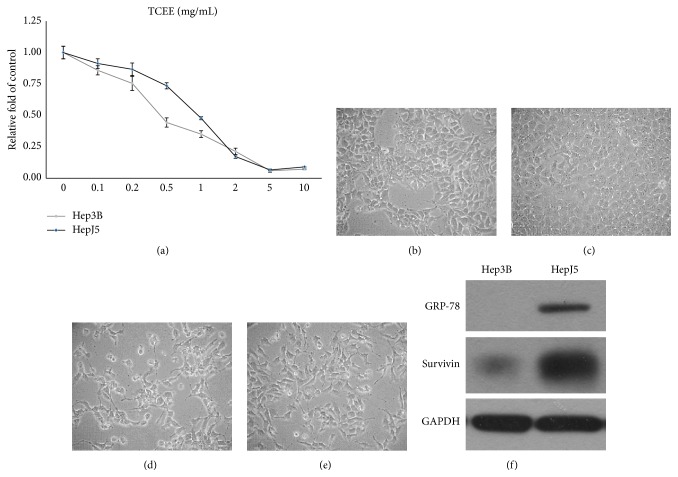
Cell growth inhibition of TCEE on human hepatocellular carcinoma cells, Hep3B and HepJ5. (a) Hep3B (gray line) and HepJ5 (black line) cells were treated with 0 to 10 mg/mL TCEE for 48 hr, and the cell viability was determined by MTT assay. IC_50_ of TCEE is 0.48 mg/mL on Hep3B cells and 0.91 mg/mL on HepJ5 cells, respectively. Experiments were repeated in triplicate and presented data were mean plus standard deviation. ((b) to (e)) Morphological observation on Hep3B and HepJ5 cells treated with 0 mg/mL TCEE ((b) and (c) Hep3B and HepJ5, resp.) or 0.5 to 1.0 mg/mL TCEE for 48 hr ((d) and (e) Hep3B and HepJ5, resp.). TCEE treated cells demonstrated apoptotic-like morphological changes such as cell shrinkage and cell blebbing compared with cells treated with normal culture medium. Magnification = 100x. (f) Expressions of survivin and GRP-78 on Hep3B and HepJ5 cells were determined by western blotting analysis. HepJ5 cells demonstrated higher expression of both survivin and GRP-78 than Hep3B cells. GAPDH served as the internal control.

For evaluating the antitumor potential of TCEE on human hepatocellular carcinoma cells, Hep3B and HepJ5 cells were treated with 0 to 10 mg/mL TCEE for 48 hr, respectively, and cell viability was analyzed by using MTT assay. As shown in Figure 1(a), TCEE significantly suppressed cell growth of both Hep3B and HepJ5 cells in a dose-dependent manner (one-way ANOVA, *P* < 0.05). The IC_50_ analysis based on the data presented in Figure 1(a) indicated that IC_50_s on Hep3B and HepJ5 were 0.48 and 0.91 mg/mL, respectively (Table 1). This result suggested that TCEE was more effective in suppressing cell growth on Hep3B rather than HepJ5 cells. In morphological observation, both Hep3B and HepJ5 cells treated with TCEE demonstrated apoptotic-like morphological changes such as cell shrinkage and cell blebbing compared with cells treated with normal culture medium (Figures 1(b)–1(e)). The overexpression of survivin and GRP-78 on HepJ5 cells was also identified by western blotting analysis (Figure 1(f)). These data together suggested that TCEE is capable of suppressing cell growth in both Hep3B and HepJ5 cells. HepJ5 cells were more resistant to TCEE treatment which may be due to the overexpression of survivin and GRP-78.

### 3.2. TCEE Induced Cell Cycle Arrest and Activation of Caspase-3 on Hepatocellular Carcinoma Cells

**Figure 2:**
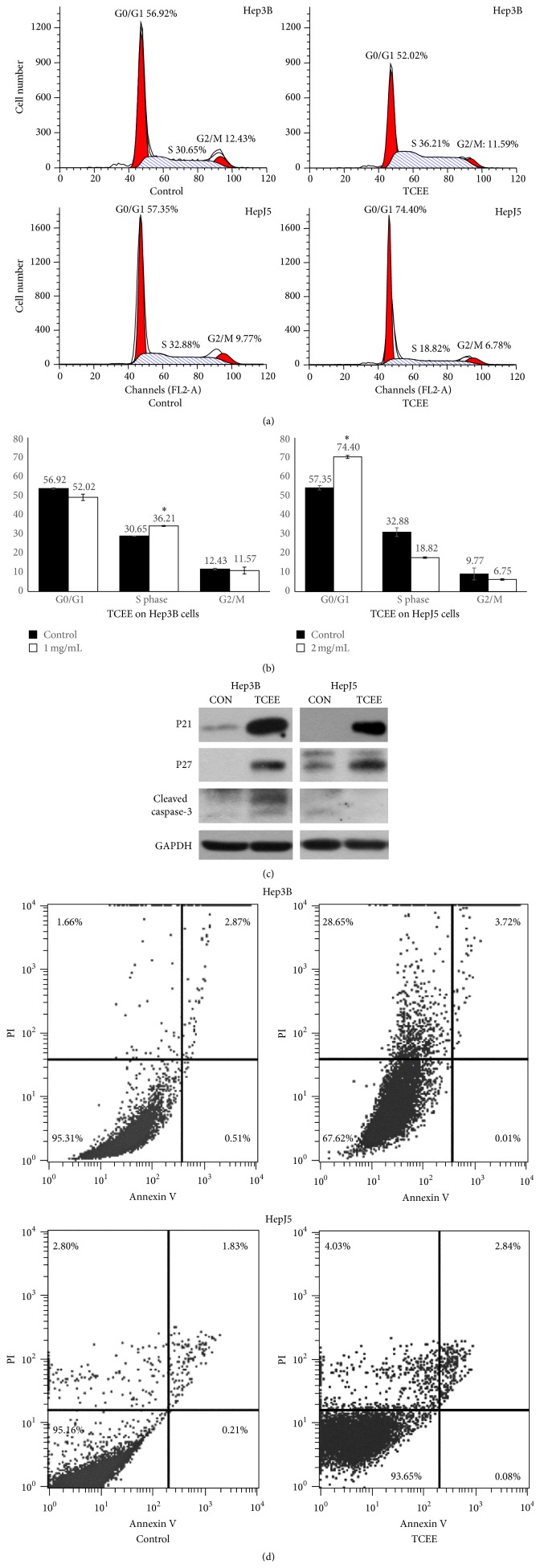
TCEE induced cell cycle arrest and activation of caspase-3. (a) The cell cycle distribution was analyzed by FACS using PI staining on Hep3B and HepJ5 cells which were treated with 1 or 2 mg/mL TCEE for 24 hr. (b) Bar charts for the comparison of control or TCEE treated cells. Data were presented as mean plus standard deviation. *∗* indicated statistical significance compared with the control group (*P* < 0.05 by Student's *t*-test). (c) Expressions of P21 and P27 and cleaved caspase-3 on Hep3B and HepJ5 cells with 0.5 or 1.0 mg/mL TCEE treatment for 48 hr were determined by western blotting analysis. GAPDH was used as the normalization control. The semiquantitative data were shown in Table 2. (d) Cell apoptosis was analyzed by the FACS analysis using the double-staining of PI and FITC-labelled annexin V on Hep3B and HepJ5 cells which were treated with 1 or 2 mg/mL TCEE for 24 hr.

**Figure 3:**
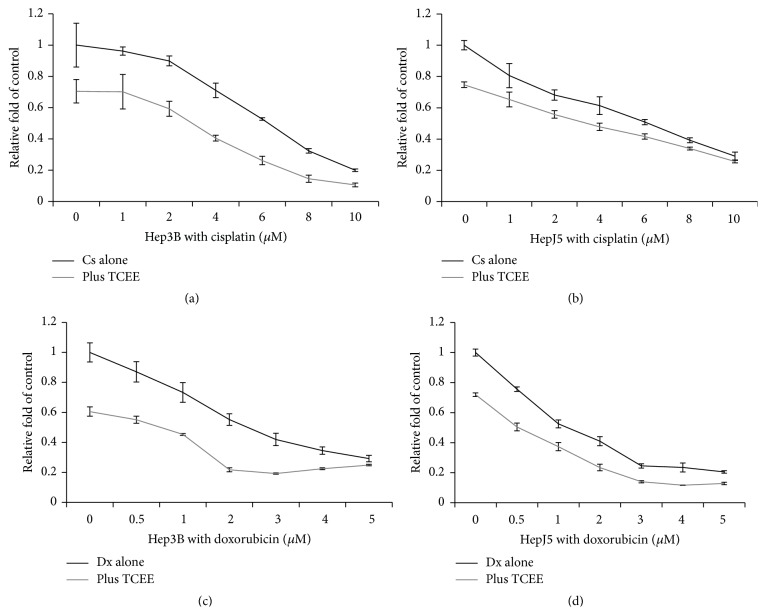
The cell growth inhibition of cisplatin or doxorubicin with TCEE treatment on Hep3B and HepJ5 cells. Hep3B and HepJ5 cells were treated with 0 to 10 *μ*M cisplatin or 0 to 5 *μ*M doxorubicin in combination with 0 (black line) or 0.2 to 0.5 mg/mL (gray line) TCEE, respectively, for 48 hr. Cell viability was determined by MTT assay. Experiments were triplicated and data were presented as mean plus standard deviation. Data were presented as mean plus standard deviation.

In order to further understand the biological mechanism of TCEE induced tumor cell suppression, Hep3B and HepJ5 cells were treated with 1 or 2 mg/mL TCEE for 24 hr and harvested for cell cycle analysis. In Figures 2(a) and 2(b), TCEE treated Hep3B cells demonstrated a larger cell distribution in S phase (36.21% versus 30.25%) whereas HepJ5 cells were in G0/G1 phase (74.40% versus 54.35%). This result suggested TCEE may lead to cell cycle arrest and are more effective on HepJ5 cells. To further identify the TCEE induced cell cycle arrest, the expressions of two cell cycle inhibitors, P21 (cyclin-dependent kinase inhibitor-1A) and P27 (cyclin-dependent kinase inhibitor-1B), were determined in TCEE treated Hep3B and HepJ5 cells.  Figure 2(c) showed that the expressions of both P21 and P27 were significantly increased by TCEE treatment. Semiquantitative data indicated that the induction folds of P21 and P27 were 36.6- and 33.2-fold on Hep3B cells, respectively, whereas on HepJ5 cells they were 85.1- and 4.5-fold (Table 2). The cleaved caspase-3, a key enzyme for programmed cell death, was also found to have a 3-fold increase on TCEE treated Hep3B cells, but not on HepJ5 cells (Table 2). Because caspase-3 is a protein marker for programed cell death including both apoptosis and necrosis, FACS analysis using the double-staining of annexin V and PI was performed to identify the TCEE induced cell death. As shown in Figure 3(d), TCEE treatment increased cell distribution on the PI-positive quadrant (upper-left) from 1.66% to 28.65% and 2.8% to 4.03% on Hep3B cells and HepJ5 cells, respectively, which indicated necrosis. Moreover, cells with both annexin V- and PI-positive staining (upper-right quadrant) were also increased from 2.87% to 3.72% and 1.83% to 2.84% on Hep3B and HepJ5 cells, respectively, which represented late apoptosis. However, there was no increased cell distribution on annexin V-positive quadrant (lower-left) which indicated the early apoptotic cells. These results suggested that TCEE treatment resulted in cell cycle arrest on both Hep3B and HepJ5 cells and also induced expression of cell cycle inhibitors, P21 and P27, on two tested cell types. Furthermore, the activation of caspase-3 was only identified in Hep3B cells. Also, double-staining of annexin V and PI indicated that TCEE may induce programmed cell death on Hep3B and HepJ5 cells, particularly necrosis on Hep3B cells.

### 3.3. TCEE Enhanced the Tumor Suppression Efficiency of Cisplatin and Doxorubicin

The results of the above study demonstrated the antitumor potential of TCEE on human hepatocellular carcinoma cells. In order to further examine the combination drug effects of TCEE with conventional chemotherapy agents, Hep3B and HepJ5 cells were treated with 0 to 10 *μ*M cisplatin or 0 to 5 *μ*M doxorubicin in combination with 0.2 or 0.5 mg/mL TCEE for 48 hr, respectively. The results of cell viability were determined by MTT assay and indicated that TCEE treatment enhanced tumor suppression efficiency of cisplatin and doxorubicin (Figure 3). Compared with the IC_50_ values on single treatment of cisplatin or doxorubicin, combination of TCEE treatment further reduced IC_50_ on Hep3B cells from 6.03 to 2.91 *μ*M and 2.43 to 0.74 *μ*M, respectively (Table 1). The enhancement of TCEE treatment with cisplatin or doxorubicin on HepJ5 cells was relatively moderate compared with Hep3B cells; IC_50_s were reduced from 5.83 to 4.27 *μ*M and 1.18 to 0.52 *μ*M (Table 1). These TCEE related combination drug effects were further analyzed by the Chou-Talalay method to obtain the combination drug index, CDI (Table 3). The CDI suggested that, on Hep3B cells, the TCEE demonstrated a synergistic effect with both cisplatin (1 to 10 *μ*M) and doxorubicin (0.5 to 5 *μ*M). In contrast, the combination drug effects of TCEE with cisplatin and doxorubicin on HepJ5 cells were between additive and synergistic effects as the CDIs were around 1. Taken together, these results suggested that TCEE enhanced tumor suppression efficiency of cisplatin and doxorubicin on human hepatocellular carcinoma cells in synergistic effect and had no antagonistic effect with the tested conventional chemotherapy agents.

## 4. Discussion

The TCEE produced from the solid-state cultivated fruit body of* T. camphoratus* is commonly used as a health supplement in Taiwan and has found antitumor potentials in human melanoma, osteosarcoma, colon, and lung cancer cells [16–18]. In this study, TCEE exhibited the antitumor capability on tested human hepatocellular carcinoma cells, Hep3B and HepJ5. In both Hep3B and HepJ5 cells, TCEE treatment induced the expression of cell cycle inhibitors, P21 and P27. Similar P21 induction by TCEE was observed in human melanoma and osteosarcoma cells [16], whereas the P27 induction by TCEE was observed in human colon cancer cells [27]. Interestingly, P21, a direct downstream protein regulated by P53, was effectively induced by TCEE treatment on the P53-deficient Hep3B cells. This result suggested an alternative pathway activated by TCEE to induce the expression of P21 on Hep3B cells. Moreover, although TCEE treatment resulted in cell cycle arrest on G0/G1 phase on HepJ5 cells but a minor level of cell cycle arrest in S phase on Hep3B cells, TCEE still demonstrated higher tumor suppression efficiency on Hep3B cells. This finding may be due to the difference of TCEE activated caspase-3 cleavage between Hep3B and HepJ5 cells. The cell death analysis using the double-staining of annexin V and PI indicated that TCEE mainly induced necrosis on Hep3B cells and partly late apoptosis in both Hep3B and HepJ5 cells. The TCEE significantly increased cleavage of caspase-3 on Hep3B cells to 2.6-fold may contribute the TCEE induced cell death on Hep3B cells, but not HepJ5 cells. Overexpression of survivin and GRP-78 on HepJ5 cells may serve as the key reason. Survivin and GRP-78 are considered as critical factors to mediate the drug resistance on HepJ5 cells with gemcitabine and sorafenib treatments [22, 28], which inhibit caspase-3 associated cell death on tumor cells [29, 30]. These results suggested the tumor suppression efficiency of TCEE on human hepatocellular cancer cells depended on individual cell features such as the expression level of survivin and GRP-78. This finding will be helpful to predict the drug response of TCEE treatment on liver cancer patients.

The organic chemical compounds with biological activity presented in* T. camphoratus* are complicated and the compositions are also very various depending on different extraction processes. The composition of TCEE is relatively well studied and known to contain diterpenoids, triterpenoids, polysaccharides, sesquiterpene lactone, and benzenoids which are considered as bioactive compounds [31]. Among these bioactive compounds presented in TCEE, 4,7-dimethoxy-5-methy-1,3-benzodioxole is suggested to suppress colon cancer cells via the activation of P53-dependent P27 pathway without induction of P21 [27], sesquiterpene lactone antrocin activates caspase-3 associated apoptosis on lung cancer cells via suppression of JAK2/STAT3 pathway [32], and also various triterpenoids activate poly(ADP-ribose) polymerase (PARP) associated cell death on prostate cancer cells [33]. These studies suggested that TCEE is a mixture with multiple antitumor compounds which suppress tumor cells via different biological mechanisms including activation of programmed cell death and regulation of cell cycle arrest. The antitumor effects of TCEE observed in Hep3B and HepJ5 cells including P21 and P27 induction and activation of caspase-3 may be due to a collective effect of these bioactive compounds.

Since TCEE demonstrated a dual-effect on suppressing human hepatocellular cancer cells by induction of cell cycle inhibitors, P21 and P27, and activation of caspase-3, it is worth it to clarify the combination effects of TCEE integrated with conventional chemotherapy agents for liver cancer. In the present study, TCEE treatment further enhanced the tumor suppression efficiency of cisplatin and doxorubicin on Hep3B and HepJ5 cells, and this enhancement was suggested to be a synergistic effect between TCEE and cisplatin/doxorubicin by the results of CDI analysis. The TCEE enhanced cisplatin/doxorubicin tumor suppression efficiency is also varied between Hep3B and HepJ5 cells. IC_50_s of cisplatin and doxorubicin were cut to half and one-third on Hep3B cells with TCEE treatment, respectively. On HepJ5 cells, TCEE cotreatment only gave a small reduction on IC_50_ of cisplatin and a half reduction with doxorubicin. These results support the previous finding that HepJ5 cells are more resistant to TCEE. Furthermore, the combination of TCEE with doxorubicin is more effective than cisplatin in both IC_50_ reduction and CDI analysis on two tested cell types. Both cisplatin and doxorubicin suppressed tumor cells by activating P21-associated cell cycle arrest and caspase-3 dependent apoptosis via caspase-8 or caspase-9 pathways [34, 35]. TCEE induced P27 expression may further promote the cell cycle arrest induced by cisplatin and doxorubicin and therefore enhance the tumor suppressing efficiency of cisplatin and doxorubicin on tumor cells. However more experimental evidences are required to clarify this possibility. On the other hand, although TCEE is considered as a safe supplement without observed toxicity on animal models [18], any unexpected adverse effects in combination of chemotherapy agents and TCEE have to be further assessed in appropriate preclinical models.

## 5. Conclusion

In this study, the antitumor potential of TCEE on human hepatocellular carcinoma cells was identified by using Hep3B and HepJ5 cells. TCEE treatment induced expression of cell cycle inhibitors, P21 and P27, as well as activation of apoptosis executer enzyme, caspase-3, on tested hepatocellular carcinoma cells. Moreover, TCEE treatment synergistically enhanced the tumor suppression efficiency of the conventional chemotherapy agents, cisplatin and doxorubicin, on Hep3B and HepJ5 cells. In conclusion, TCEE is a potential ingredient which may be integrated with cisplatin- or doxorubicin-based chemotherapy for treating liver cancer patients. More preclinical researches are therefore required to verify the clinical performance of TCEE with the conventional chemotherapy.

## Figures and Tables

**Table 1:** Half-maximal inhibitory concentration (IC_50_) analysis on Hep3B and HepJ5 cells treated with TCEE alone or in combination with cisplatin or doxorubicin. The IC_50_ values of TCEE on Hep3B and HepJ5 were analyzed on cell viability data obtained from Figure 1 by using the CalcuSyn software, and the IC_50_ values of cisplatin and doxorubicin with TCEE were from Figure 3.

	Hep3B		HepJ5	
TCEE (mg/mL)	0.48		0.91	

Combined treatment with conventional chemotherapy agents				
	TCEE (mg/mL)			
	0	0.2	0	0.5

Cisplatin (*μ*M)	5.38	2.29	4.57	2.82
Doxorubicin (*μ*M)	2.37	0.91	1.28	0.51

**Table 2:** Semiquantitative analysis of TCEE induced P21 and P27 expression and cleavage of caspase-3 on Hep3B and HepJ5 cells. Data were presented as mean plus standard deviation of induction fold compared with Hep3B and HepJ5 cells treated with normal culture serum. The presented values were relative induction fold of P21 and P27 and cleaved caspase-3 on TCEE treated cells compared with normal culture medium treated cells. Experiments were repeated in triplicate. *P* values were determined by Student's *t*-test. Semiquantitative data were normalized by expression of GAPDH.

	Hep3B	*P*	HepJ5	*P*
P21	36.6 ± 13.4	<0.01	85.1 ± 6.1	<0.01
P27	33.2 ± 8.5	<0.01	4.5 ± 1.1	<0.01
Cleaved caspase-3	2.6 ± 0.4	<0.01	1.4 ± 1.2	0.63

**Table 3:** Analysis of combination drug effects of TCEE with cisplatin or doxorubicin in Hep3B and HepJ5 cells. Combination drug index (CDI) was analyzed on cell viability data obtained from Figure 3 by using the CalcuSyn software. CDI < 1 indicated a synergistic effect, CDI = 1 indicated an additive effect, and CDI > 1 indicated an antagonistic effect.

		Combination drug index	
		Hep3B	HepJ5
		Plus 0.2 mg/mL TCEE	Plus 0.5 mg/mL TCEE
Cisplatin (*μ*M)	1	0.52	0.75
	2	0.59	0.77
	4	0.64	0.96
	6	0.61	1.08
	8	0.48	1.03
	10	0.47	0.88
	20	1.16	0.55

Doxorubicin (*μ*M)	0.5	0.61	1.39
	1	0.77	1.04
	1.5	0.74	0.71
	2	0.38	0.79
	3	0.32	0.72
	4	0.58	0.58
	5	0.67	0.78
